# High Prevalence of Carbapenemase-Producing *Acinetobacter baumannii* in Wound Infections, Ghana, 2017/2018

**DOI:** 10.3390/microorganisms9030537

**Published:** 2021-03-05

**Authors:** Mathieu Monnheimer, Paul Cooper, Harold K. Amegbletor, Theresia Pellio, Uwe Groß, Yvonne Pfeifer, Marco H. Schulze

**Affiliations:** 1 Institute for Medical Microbiology and Göttingen International Health Network, University Medical Center Göttingen, Kreuzbergring 57, 37075 Göttingen, Germany; Mathieu.Monnheimer@gmx.net (M.M.); ugross@gwdg.de (U.G.); 2 St. Martin de Porres Hospital, Post Office Box 06, Eikwe via Axim, Ghana; cpaulkofi@yahoo.co.uk (P.C.); haroldam@yahoo.com (H.K.A.); tpellio@gmx.de (T.P.); 3 Nosocomial Pathogens and Antibiotic Resistance, Robert Koch Institute, Burgstrasse 37, 38855 Wernigerode, Germany; pfeifery@rki.de; 4 Institute of Infection Control and Infectious Diseases, University Medical Centre Göttingen, Robert-Koch-Strasse 40, 37075 Göttingen, Germany

**Keywords:** *Acinetobacter baumannii*, carbapenem resistance, OXA-23, OXA-420, NDM-1, wound infections, Ghana, rural

## Abstract

Three years after a prospective study on wound infections in a rural hospital in Ghana revealed no emergence of carbapenem-resistant bacteria we initiated a new study to assess the prevalence of multidrug-resistant pathogens. Three hundred and one samples of patients with wound infections were analysed for the presence of resistant bacteria in the period August 2017 till March 2018. Carbapenem-resistant *Acinetobacter (A.) baumannii* were further characterized by resistance gene sequencing, PCR-based bacterial strain typing, pulsed-field gel electrophoresis (PFGE) and multilocus sequence typing (MLST “Oxford scheme”). *A. baumanni* was detected in wound infections of 45 patients (15%); 22 isolates were carbapenem-resistant. Carbapenemases NDM-1 and/or OXA-23 were detected in all isolates; two isolates harboured additionally OXA-420. PFGE and MLST analyses confirmed the presence of one *A. baumannii* strain in 17 patients that was assigned to the worldwide spread sequence type ST231 and carried NDM-1 and OXA-23. Furthermore, two new *A. baumannii* STs (ST2145 and ST2146) were detected in two and three patients, respectively. Within three years the prevalence of carbapenem-resistant *A. baumannii* increased dramatically in the hospital. The early detection of multidrug-resistant bacteria and prevention of their further spread are only possible if continuous surveillance and molecular typing will be implemented.

## 1. Introduction

Antibiotics are essential medicines whose use in human or veterinary medicine, no matter how prudent, is inevitably associated with accelerated development of antimicrobial resistance (AMR). β-lactams are still considered the most successful antibiotic classes. With a proportion of almost two thirds of all antibiotic prescriptions they are even the most widely used antibacterial agents against infectious diseases [1]. The carbapenems have the broadest spectrum of activity against various bacteria and are widely regarded as the class of last resort for treatment of infections with multidrug-resistant pathogens [2]. However, resistance to carbapenems has increased dramatically worldwide [3]. One reason for this resistance is the production of different carbapenem-hydrolyzing enzymes, the carbapenemases. In the last 20 years, the number of newly detected carbapenemases has increased continuously. The most prevalent carbapenemases in *Enterobacterales* are OXA-48, KPC, VIM and NDM; in *Pseudomonas (P.) aeruginosa* VIM and IMP have been frequently detected, and different OXA-enzymes (OXA-23, OXA-58, OXA-40-like) and NDM have been found mainly in *Acinetobacter (A.) baumannii* [3,4,5,6]. The location of carbapenemase genes within transposons and/or conjugative plasmids enables the emergence in various bacterial species and facilitates their worldwide spread [7,8].

Treatment of infections caused by carbapenem-resistant pathogens is quite a big challenge in industrialized countries and almost impossible for health systems with limited resources [9,10,11]. Of great concern are carbapenemase-producing *A. baumannii*, *P. aeruginosa* and *Enterobacterales* [12,13,14]. Remaining treatment option for *Enterobacterales* producing serine carbapenemases, e.g., KPC or OXA-48, might be ceftazidime-avibactam, meropenem-vaborbactam, imipenem-cilastatin-relebactam, or cefiderocol; for *Enterobacterales* producing metallo-beta-lactamases, e.g., NDM, aztreonam plus ceftazidim-avibactam or cefiderocol. Alternatively, colistin (plus meropenem or tigecycline or eravacycline) could be an option. Therapeutic options for carbapenem-resistant *A. baumannii* might be cefiderocol or colistin (plus a carbapenem, minocycline, tigecycline, or rifampicin); for carbapenem-resistant *P. aeruginosa* ceftolozane-tazobactam, imipenem-cilastatin-relebactam, cefiderocol or colistin plus meropenem [15]. However, their availability especially in low- and middle-income countries is rather unlikely [16].

*A. baumannii* is a non-fermenting bacterium of importance in both humid and temperate climates [17]. Colonization of the human skin may lead to community-acquired or nosocomial traumatic or surgical wound infections [18,19]. However, the distinction between true infection and colonization is often difficult to decide [20]. Wound infections are a common disease entity that is frequently not given the necessary attention, including lack of microbiological investigation, leading to chronicity and disability [21]. Biofilms are present in most chronic wounds and may contribute to delayed healing and persistent inflammation. The environment of the biofilm facilitates the horizontal spread of antibiotic resistance genes and virulence factors between embedded pathogenic bacteria [22]. Moreover, bacteria are protected from external threats creating additional bacterial tolerance to antimicrobial agents [23]. Microbiological culture of wound specimens performed through deep swabbing techniques and processed within two hours frequently detects a polymicrobial flora. Deciding which of these different pathogens should be treated is always a big challenge for the clinician.

Prevention and control of multidrug-resistant bacteria [24] and availability of up to date recommendations for antimicrobial therapy [25] require continuous surveillance of antimicrobial resistance which is still not established in most Sub-Saharan African countries [26]. A pilot study by our study group in a rural hospital in Eikwe, Western Region of Ghana, found a high prevalence of *Enterobacterales* with combined resistance to third-generation cephalosporins and fluoroquinolones but no carbapenem-resistant bacteria at all in wound infections [27]. The results of this pilot study in 2014 prompted the project partners in Eikwe and Göttingen to conduct another study. Therefore, a more comprehensive microbiological analysis of wound infections in Ghana was carried out from August 2017 to March 2018. All detected carbapenem-resistant *A. baumannii* were characterized in the present study.

## 2. Materials and Methods

### 2.1. Ethics

The study was approved by the Ghana Health Service Ethics Review Committee, Research & Development Division, Ghana Health Service, Accra, Ghana (GHS-ERC: 04/06/17, 21 July 2017), and the Ethics Committee of the University Medical Center in Göttingen, Germany (5/6/17, 20 June 2017), respectively. All patients provided written informed consent before inclusion in the study.

### 2.2. Clinical Diagnosis and Sample Collection

Wound infection was diagnosed clinically using the classic signs of inflammation. Before taking the sample, wounds were cleaned with sterile cotton swabs moistened with sterile sodium chloride solution (0.9%). The sample was taken with an eSwab^TM^ (Copan, Brescia, Italy) from the wound bed and edge and then immediately brought to the bacteriology laboratory for further processing.

### 2.3. Microbiological Culture and Susceptibility Testing

Basic biochemical identification of the bacterial isolates and antibiotic susceptibility testing were initially carried out in the bacteriology laboratory of the hospital in Eikwe adapted to the locally available resources. The anti-infective treatment of wound infections was adjusted according to the results of the microbiological analysis as described before [27]. In brief, samples were inoculated on 5% blood agar (Merck, Darmstadt, Germany) and on McConkey agar (Merck, Darmstadt, Germany), incubated aerobically at 37 °C, and read after 24 h and 48 h. Gram-negative bacteria were identified through: (i) hydrogen sulphide production, indole production and motility in semi-solid SIM (sulphide, indole, motility) medium (Merck, Darmstadt, Germany) vertically in tubes; (ii) sucrose fermentation in Hugh Leifson medium (Merck, Darmstadt, Germany); and (iii) Oxidase test, respectively. With this local approach, identification down to the genus level was possible. The microscopical examination of a Gram-stained smear was done in order to ensure wound specimen quality and to check for presence of bacteria, neutrophils, and epithelial cells. Furthermore, disc diffusion tests for Gram-negative bacteria were performed using the following paper antibiotic discs: ampicillin 10 µg; ampicillin-sulbactam 10 µg–10 µg; cefotaxime 5 µg; ciprofloxacin 5 µg; gentamicin 10 µg; and trimethoprim-sulfamethoxazole 1.25–23.75 µg, respectively [28]. There was no routine screening for carbapenem susceptibility.

All bacterial isolates, independent of their antibiotic resistance, were stored at −20 °C in microbank systems, and were reanalysed at the Institute for Medical Microbiology of the University Medical Center in Göttingen, Germany, and at the Robert Koch Institute in Wernigerode, Germany. Species identification was done with MALDI Biotyper 3.0 (Bruker Daltonics, Bremen, Germany). Antibiotic susceptibility testing for *A. baumannii* isolates was performed by broth microdilution and VITEK 2 (bioMérieux, Marcy-l’Étoile, France) using card AST-N248 (bioMérieux, Nuertingen, Germany) with interpretation of results according to EUCAST criteria (EUCAST v10.0).

### 2.4. Molecular Analyses

For all *A. baumannii* isolates with resistance to meropenem and/or imipenem the presence of intrinsic *bla*_OXA-51-like_ genes and the presence of different carbapenemase and other β-lactamase genes (IS*Aba1*+*bla*_OXA-51-like_, *bla*_OXA-23-like_, *bla*_OXA-24-like_, *bla*_OXA-58-like_, *bla*_IMP-like_, *bla*_VIM-like_, *bla*_NDM-like_, *bla*_GES-like_, *bla*_PER-like_, *bla*_VEB-like_) was investigated by PCR and Sanger sequencing, as described previously [29,30,31]. Furthermore, all carbapenem-resistant *A. baumannii* were tested for assignment to the important international clones 1–3 (formerly named European clones I–III) by multiplex-PCR [32]. Bacterial strain typing was done by *Apa*l-macrorestriction and subsequent pulsed-field gel electrophoresis (PFGE) and results were interpreted according to the criteria of Tenover et al. [33]. Finally, multilocus sequence typing (MLST) was performed for selected isolates (PFGE types A-1, A-2, A-3) according to the “Oxford” scheme (https://pubmlst.org/organisms/acinetobacter-baumannii, accessed on 3 August 2020).

## 3. Results

### 3.1. Wound Infection Classification

Overall, wound swabs from 301 patients with wound infections were analysed. Wound infection (WI) (duration of infection ≤ three month), chronic wound infection (CWI) (duration of infection > three month), and surgical site infection (SSI) was diagnosed in 144 (48%), 70 (23%), and 74 (25%) patients, respectively. No information was available in 13 (4%) patients.

### 3.2. Identification and Susceptibility of Detected Bacterial Pathogens

**Figure 1:**
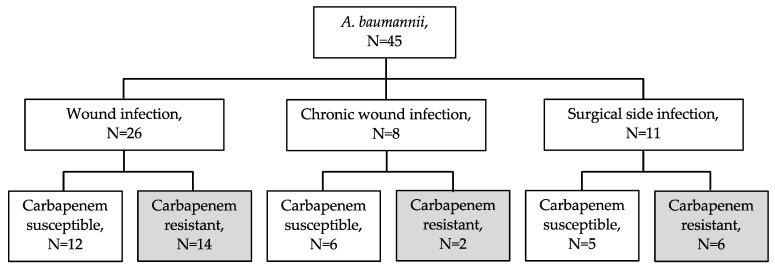
Distribution of *Acinetobacter baumannii* isolates according to wound infection type and carbapenem susceptibility.

*A. baumannii* was isolated in wound swabs of 45 patients. Carbapenem resistance was detected in 22 (49%) of these 45 *A. baumannii* isolates, Figure 1. Further bacterial pathogens which were detected beside these carbapenem-resistant *A. baumannii* in wound swabs are listed in Table 1. However, no other carbapenem-resistant bacteria except three *Stenotrophomonas maltophilia* and one *P. aeruginosa* without transmissible carbapenemase genes were found. The 22 carbapenem-resistant *A. baumannii* isolates were additionally resistant to ciprofloxacin, trimethoprim-sulfamethoxazole, gentamicin and amikacin (20 of 22 isolates) but remained susceptible to colistin (Table 2).

### 3.3. Molecular Characteristics of A. baumannii

The majority of the 22 carbapenem-resistant *A. baumannii* isolates (*n* = 17, 77%) harboured the two carbapenemase genes *bla*_OXA-23_ and *bla*_NDM-1_. In two isolates the combination *bla*_NDM-1_ and *bla*_OXA-420_ was detected. The three remaining isolates carried *bla*_OXA-23_, and the insertion sequence IS*Aba1* was present upstream of the intrinsic gene *bla*_OXA-378_ (Table 2).

By PCR-based typing the 17 *A. baumannii* isolates with carbapenemase gene combination *bla*_OXA-23_/*bla*_NDM-1_ were assigned to international clone 1 (IC 1). All 17 isolates harboured *bla*_OXA-69_, a variant of the intrinsic, *A. baumannii* specific *bla*_OXA-51_ gene. The five remaining isolates were non-typeable by this multiplex-PCR and carried the intrinsic gene variant *bla*_OXA-378_.

**Figure 2:**
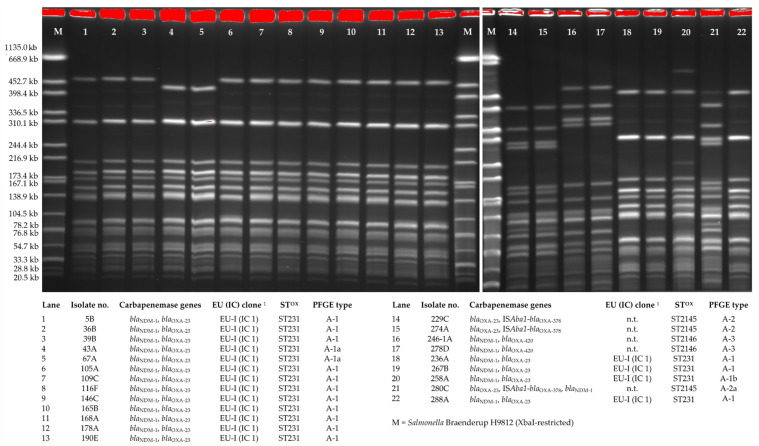
ApaI-macrorestriction and pulsed-field gel electrophoresis of 22 carbapenem-resistant *A. baumannii* isolates from wound infections, Ghana, 2017/2018. **^1^** PCR Turton et al. 2007 [32]. ST^OX^, sequence type assigned by multilocus sequence typing (MLST Oxford scheme https://pubmlst.org/organisms/acinetobacter-baumannii, assessed on 3 August 2020); n.t. non-typeable.

PFGE-typing of the 22 carbapenemase-producing isolates revealed three distinctly distinguishable macrorestriction patterns designated as *A. baumannii* PFGE-types A-1, A-2 and A-3 (Figure 2). The 17 *A. baumannii* isolates of IC 1 were assigned to PFGE-type A-1 and showed highly related macrorestriction patterns that differed in 0–2 bands. PFGE-type A-2 isolates (*n* = 3) differed in six bands from PFGE-type A-3 isolates (*n* = 2) but all five isolates carried the intrinsic gene *bla*_OXA-378._

Using MLST (“Oxford” scheme) the 17 *A. baumanni* isolates of IC 1 were assigned to sequence type ST231 (Table 2). The five other isolates were designated as new sequence types ST2145 and ST2146, respectively.

## 4. Discussion

The molecular analyses of the 22 detected carbapenem-resistant *A. baumannii* isolates in this study confirmed the presence of an NDM-1 and OXA-23 carbapenemase-producing strain of sequence type ST231 in wounds of 17 patients. According to the MLST database ST231 (Table 2) has been detected for clinical isolates worldwide, e.g., Brasilia, Libya, the USA, the Netherlands and Germany [29]. The emergence and spread of this carbapenemase-producing strain of ST231 within the hospital in Eikwe is of concern and needs continuous surveillance.

Furthermore, three and two patients carried *A. baumannii* of the novel sequence types ST2145 and ST2146, respectively. Both STs differed in only one allele (*gpi*) to ST1452 and ST1459, that were detected previously for several isolates with *bla*_OXA-378_ from white stork nestlings in Poland, 2015 [34]. The strain of ST2145 carried carbapenemase gene *bla*_OXA-23_ and insertion sequence IS*Aba1* upstream of *bla*_OXA-378_ that is known to provide a strong promoter for *bla*_OXA-51-like_ genes in *A. baumannii* resulting in increased gene expression and resistance to carbapenems [31]. For the strain of ST2146 carbapenemase genes *bla*_NDM-1_ and *bla*_OXA-420_ were detected. OXA-420 has been reported first in carbapenem-resistant *A. baumannii* isolates from Nepal in 2014 and it shows one amino acid substitution (A256D) compared to the worldwide prevalent carbapenemase OXA-58 [35]. Furthermore, the *bla*_OXA-420_ gene sequence has been submitted to the NCBI database from *A. baumannii* isolates in the Netherlands (CP038646.1, April 2019); and the genome analysis of 36 *A. baumannii* from three hospitals in Ghana (2016–2017) revealed one ST107 isolate from a sputum sample with this rare enzyme variant [36]. Since carbapenemase genes are often located on plasmids a transfer between different *A. baumannii* strains might be possible but this was not analyzed further in the present study.

Our pilot study in Eikwe, Western Region of Ghana, 2014 [27], and a study from another rural district hospital in Asante Akim North Municipality of Ghana, 2016 [37] found no carbapenem-resistant *A. baumannii*. However, three and a half years later, the present study has shown that almost half of the *A. baumannii* isolates from wound infections are carbapenem-resistant. Several circumstances might have been contributed. Exposure to carbapenems is a necessary but not sufficient prerequisite. Carbapenems are available in Ghana, but their clinical use seems to be very limited and most likely restricted to very selected patients in private or university hospitals [38]. The increasing mobility of local people nationally and even internationally for private and/or professional reasons could lead to the acquisition of carbapenem-resistant *A. baumannii* isolates [39,40,41]. In addition, colonized patients admitted to multiple health facilities might contribute to the further spread of opportunistic bacteria. Once carbapenem-resistant *A. baumannii* is introduced into a local health facility and there is no continuous microbiological surveillance to detect the phenomenon of carbapenem resistance, together with inadequate infection control measures the gateway to further transmission is open.

The treatment of patients with carbapenem-resistant *A. baumannii* wound infection is difficult. There is no oral treatment option available. Colistin might be therapeutic options but in resource-limited countries such as Sub-Saharan Africa, these drugs are simply not available or too expensive [42]. Ultimately, only wound debridement, use of antiseptics which favour the formation of granulation tissue and appropriate wound care with careful adherence to hygiene standards are realistic options. Therefore, investment in and continuous training of horizontal infection control measures like appropriate hand hygiene, e.g., WHO 5 moments of hand hygiene, need to be promoted and trained continuously [43].

## 5. Conclusions

In conclusion, the emergence and nosocomial spread of carbapenem-resistant *A. baumannii* in this follow up investigation in Ghana demonstrates that continuous surveillance of antimicrobial resistance of clinical isolates is of paramount importance. Health systems with limited resources need to be enabled to perform nationwide basic microbiological investigations on a routine basis. However, there is also need to get access to more sophisticated technologies like molecular typing methods to analyse emergence and spread of multidrug-resistant bacteria and to take immediate and appropriate preventive hygienic measures. Finally, access to effective anti-infective drugs according to the local resistance situation is urgently needed.

## Figures and Tables

**Table 1:** List of bacterial pathogens detected beside carbapenem-resistant *Acinetobacter baumannii* in wound infections (*n* = 22).

Co-Detected Bacterial Species in Wound Infections (*n* = Number of Wound Infections)	
Gram-Positive Pathogens	Gram-Negative Pathogens
*Staphylococcus aureus*, *n* = 6	*Proteus mirabilis*, *n* = 6
*Corynebacterium striatum*, *n* = 3	*Escherichia coli*, *n* = 4
*Bacillus cereus*, *n* = 2	*Stenotrophomonas maltophilia*, *n* = 3
*Corynebacterium amycolatum*, *n* = 1	*Enterobacter cloacae*, *n* = 2
*Enterococcus avium*, *n* = 1	*Klebsiella pneumoniae*, *n* = 2
*Enterococcus casseliflavus*, *n* = 1	*Pseudomonas aeruginosa*, *n* = 2
*Enterococcus faecalis*, *n* = 1	*Achromobacter spanius*, *n* = 1
	*Providencia stuartii*, *n* = 1

Between 0 and 5 further bacterial pathogens beside carbapenem-resistant *A. baumannii* were detected in the individual wound infection. The average number of pathogens per wound infection was 2.7. Coagulase-negative staphylococci (*n* = 7) other than *Staphylococcus lugdunensis* or *Staphylococcus schleiferi* subsp. *schleiferi* were present but not further analysed.

**Table 2:** Characteristics of 22 carbapenem-resistant *Acinetobacter baumannii* isolates from wound infections, Ghana, 2017/2018.

Isolate No.	Sampling Date	Species	*A. baumannii* Specific *bla*_OXA-51-like_^1^	Carbapenemase Genes	*A. baumannii* Typing PCR (clones EI-III) ^2^	PFGE-Type	Sequence Type (ST)^OX^	Resistances ^3^
5B	21.08.2017	*A. baumannii*	*bla*_OXA-69_	*bla*_NDM-1_, *bla*_OXA-23_	EU-I (IC 1)	A-1	ST231	IPM, MEM, GEN, AMK, CIP, SXT
36B	22.09.2017	*A. baumannii*	*bla*_OXA-69_	*bla*_NDM-1_, *bla*_OXA-23_	EU-I (IC 1)	A-1	ST231	IPM, MEM, GEN, AMK, CIP, SXT
39B	26.09.2017	*A. baumannii*	*bla*_OXA-69_	*bla*_NDM-1_, *bla*_OXA-23_	EU-I (IC 1)	A-1	ST231	IPM, MEM, GEN, AMK, CIP, SXT
43A	27.09.2017	*A. baumannii*	*bla*_OXA-69_	*bla*_NDM-1_, *bla*_OXA-23_	EU-I (IC 1)	A-1a	ST231	IPM, MEM, GEN, AMK, CIP, SXT
67A	12.10.2017	*A. baumannii*	*bla*_OXA-69_	*bla*_NDM-1_, *bla*_OXA-23_	EU-I (IC 1)	A-1a	ST231	IPM, MEM, GEN, AMK, CIP, SXT
105A	06.11.2017	*A. baumannii*	*bla*_OXA-69_	*bla*_NDM-1_, *bla*_OXA-23_	EU-I (IC 1)	A-1	ST231	IPM, MEM, GEN, AMK, CIP, SXT
109C	08.11.2017	*A. baumannii*	*bla*_OXA-69_	*bla*_NDM-1_, *bla*_OXA-23_	EU-I (IC 1)	A-1	ST231	IPM, MEM, GEN, AMK, CIP, SXT
116F	10.11.2017	*A. baumannii*	*bla*_OXA-69_	*bla*_NDM-1_, *bla*_OXA-23_	EU-I (IC 1)	A-1	ST231	IPM, MEM, GEN, AMK, CIP, SXT
146C	27.11.2017	*A. baumannii*	*bla*_OXA-69_	*bla*_NDM-1_, *bla*_OXA-23_	EU-I (IC 1)	A-1	ST231	IPM, MEM, GEN, AMK, CIP, SXT
165B	06.12.2017	*A. baumannii*	*bla*_OXA-69_	*bla*_NDM-1_, *bla*_OXA-23_	EU-I (IC 1)	A-1	ST231	IPM, MEM, GEN, AMK, CIP, SXT
168A	11.12.2017	*A. baumannii*	*bla*_OXA-69_	*bla*_NDM-1_, *bla*_OXA-23_	EU-I (IC 1)	A-1	ST231	IPM, MEM, GEN, AMK, CIP, SXT
178A	15.12.2017	*A. baumannii*	*bla*_OXA-69_	*bla*_NDM-1_, *bla*_OXA-23_	EU-I (IC 1)	A-1	ST231	IPM, MEM, GEN, AMK, CIP, SXT
190E	22.12.2017	*A. baumannii*	*bla*_OXA-69_	*bla*_NDM-1_, *bla*_OXA-23_	EU-I (IC 1)	A-1	ST231	IPM, MEM, GEN, AMK, CIP, SXT
229C	15.01.2018	*A. baumannii*	*bla*_OXA-378_	*bla*_OXA-23_, IS*Aba1*-*bla*_OXA-378_	n.t.	A-2	ST2145	IPM, MEM, GEN, CIP, SXT
236A	17.01.2018	*A. baumannii*	*bla*_OXA-69_	*bla*_NDM-1_, *bla*_OXA-23_	EU-I (IC 1)	A-1	ST231	IPM, MEM, GEN, AMK, CIP, SXT
246-1A	24.01.2018	*A. baumannii*	*bla*_OXA-378_	*bla*_NDM-1_, *bla*_OXA-420_	n.t.	A-3	ST2146	IPM, MEM, GEN, AMK, CIP, SXT
258A	05.02.2018	*A. baumannii*	*bla*_OXA-69_	*bla*_NDM-1_, *bla*_OXA-23_	EU-I (IC 1)	A-1b	ST231	IPM, MEM, GEN, AMK, CIP, SXT
267B	06.02.2018	*A. baumannii*	*bla*_OXA-69_	*bla*_NDM-1_, *bla*_OXA-23_	EU-I (IC 1)	A-1	ST231	IPM, MEM, GEN, AMK, CIP, SXT
274A	14.02.2018	*A. baumannii*	*bla*_OXA-378_	*bla*_OXA-23_, IS*Aba1*-*bla*_OXA-378_	n.t.	A-2	ST2145	IPM, MEM, GEN, CIP, SXT
278D	19.02.2018	*A. baumannii*	*bla*_OXA-378_	*bla*_NDM-1_, *bla*_OXA-420_	n.t.	A-3	ST2146	IPM, MEM, GEN, AMK, CIP, SXT
280C	19.02.2018	*A. baumannii*	*bla*_OXA-378_	*bla*_OXA-23_, IS*Aba1*-*bla*_OXA-378_, *bla*_NDM-1_	n.t.	A-2a	ST2145	IPM, MEM, GEN, AMK, CIP, SXT
288A	24.02.2018	*A. baumannii*	*bla*_OXA-69_	*bla*_NDM-1_, *bla*_OXA-23_	EU-I (IC 1)	A-1	ST231	IPM, MEM, GEN, AMK, CIP, SXT

^1^ PCR Turton et al. 2006 [31]. ^2^ PCR Turton et al. 2007 [32]. n.t. non-typeable. ^3^ All carbapenem-resistant *A. baumannii* isolates were susceptible to colistin. ST^OX^, sequence type according multilocus sequence typing (MLST “Oxford” scheme (https://pubmlst.org/organisms/acinetobacter-baumannii, accessed on 3 August 2020). IPM, imipenem; MEM, meropenem; GEN, gentamicin; AMK; amikacin; CIP, ciprofloxacin; SXT, trimethoprim-sulfamethoxazole.

## Data Availability

The information on two *A. baumanni* isolates with novel sequence types ST2145 (no. 71-20, ID5021) and ST2146 (no. 73-20, ID5022) was submitted to the MLST database (https://pubmlst.org/bigsdb?db=pubmlst_abaumannii_isolates, assessed on 3 August 2020).
